# Peripheral arterial elasticity changes derived by volume-oscillometry in reaction to hyperemia as a possible assessment of flow-mediated vasodilatation

**DOI:** 10.1038/s41598-022-22050-1

**Published:** 2022-11-14

**Authors:** Takehiro Yamakoshi, Peter Rolfe, Ken-ichi Yamakoshi

**Affiliations:** 1 Institute of Liberal Arts and Science, Kanazawa University, Kakuma-machi, Kanazawa, Ishikawa 920-1192 Japan; 2 Department of Research and Development, Nonprofit Organization of Research Institute of Life Benefit, 6-11-7-1 Sumikawa, Minami-ku, Sapporo, Hokkaido 005-0006 Japan; 3 Department of Automatic Measurement and Control, Harbin Institute of Technology, No. 92 West Dazhi Street, Nan Gang District, Harbin, 150001 Heilongjiang Province China; 4 Science and Technology, Oxford BioHorizons Ltd., 23 West Bar St., Banbury, OX16 9SA Oxfordshire UK; 5 College of Science and Engineering, Kanazawa University, Kakuma-machi, Kanazawa, Ishikawa 920-1192 Japan; 6 Department of Orthopaedic Surgery, Showa University School of Medicine, 1-5-8 Hatanodai, Shinagawa-ku, Tokyo, 142-8555 Japan; 7 Nonprofit Organization of Research Institute of Life Benefit, 6-11-7-1 Sumikawa, Minami-ku, Sapporo, Hokkaido 005-0006 Japan

**Keywords:** Predictive medicine, Cardiovascular biology, Disease prevention

## Abstract

The flow-mediated dilation (FMD) test is commonly utilized and is the only technique for the assessment of vascular endothelial cell function. With this test, the augmentation of a brachial artery diameter following reactive hyperemia is measured precisely using ultrasonography by a skilled operator. This is a hospital-only test, and would be more useful if conveniently performed at home. This paper describes a first approach for studying the impact of changes in peripheral arterial elasticity, with prospects towards possible assessment of functional reactivity. A recently developed smartphone-based instrument was used to measure elastic properties of finger and radial arteries, related to stiffness and vasodilatation, as a function of distending pressure derived by photo-plethysmographic volume-oscillometry. Elasticity changes in both arteries before and after a 5-min supra-systolic upper-arm cuff occlusion were successfully obtained in 15 normal volunteers. The index-values of stiffness and vasodilatation showed, respectively, a significant decrease and increase (*p* < 0.01), demonstrating clearly the expected elasticity changes with hyperemia, which could be consistent with the clinically-stated reaction in an FMD test. The results suggest that this method could easily provide important information of both elasticity and vasodilatation. It appears promising as a convenient assessment method to contribute to arteriosclerotic cardiovascular screening.

## Introduction

Due to the rapid growth in the super-aging population worldwide, an increasing number of patients have been suffering from such cardiovascular related diseases as myocardial and cerebral infarction, the major cause of which is arteriosclerosis, well known as a “silent killer”^1,2^. Prevention, early diagnosis and treatment of vascular dysfunction as well as cardiovascular complications are therefore of great importance; noninvasive methods are preferred, where possible. Currently, there are several noninvasive vascular function tests performed in clinical practice, such as flow-mediated dilation (FMD)^3–5^, pulse wave velocity (PWV)^6,7^, ankle-brachial pressure index (ABI)^8^ and carotid echocardiography^9^. However, almost all of these tests cannot provide any specific information about arterial mechanical properties themselves, despite their importance and usefulness in clinical practice. This is due mainly to the technical difficulty of quantitatively acquiring representative measures of mechanical properties.

Among these function tests, the FMD test is at least commonly utilized and, in fact, is the only assessment technique for the acquisition of vascular endothelial function. The latter is one of the most important causative factors of arteriosclerotic cardiovascular diseases, and is considered to be a possible potential factor in producing future cardiovascular disease events^3–5,10^. Additionally, it is noteworthy that vasodilatory functions can be strongly associated with peripheral circulatory injuries. One important clinical problem can be the creation of pressure ulcers and the tests can be useful here as part of a treatment to improve sacral skin blood circulation, which is especially important for people with spinal cord injury^11–13^.

With the FMD test, the inner diameter of a brachial artery is measured using ultrasonic equipment before (baseline) and after a 5-min supra-systolic cuff occlusion of the forearm. Vasodilatation after release of the cuff occlusion occurs following an acute increase in blood flow, typically induced via circulatory arrest in the arm. This hyperemia increases shear stress of the inner vessel wall which is transferred to the endothelial cells, inducing nitric oxide production (well known as an endothelium-derived vascular relaxation factor), and then a subsequent vasodilatation occurs^4^. The increase in arterial diameter, as a consequence of the reactive hyperemia, is compared to the baseline diameter and expressed simply as a percentage of this baseline diameter (%_FMD), which is clinically revealed to be more than 6–7% (up to around 25%) in normal young and old subjects^3,10^.

Although the FMD test can have clinical utility, there are several technical and operational issues to be addressed in terms of more precise and continuous measurement of arterial diameter, more appropriate attachment of an ultrasonic probe, more compact ultrasonic device with greater portability^3,14^. This is a hospital-only test, and it would be more desirable and useful for a person to perform a test similar to FMD by him-/herself without the use of ultrasonography, at home as with blood pressure measurement. This would allow checking the vascular function at any time, and even if the subject has an early arteriosclerosis such home-based check could be helpful for a follow-up examination to introduce lifestyle improvements and/or drug therapeutic measures to manage the vascular condition. In this connection a convenient method to evaluate the FMD without using an ultrasound technique was developed, named as “enclosed zone FMD (ezFMD)”^15,16^. This was based on a conventional cuff-oscillometric sphygmomanometer to predict vasodilatation of the radial artery by measuring cuff-pressure oscillations. Although this method is easy and appropriate to use at home, it is difficult to acquire quantitatively direct radial arterial diameter movements as well as arterial mechanical properties.

Taking the vasodilatation mechanism into account, as mentioned above, it is naturally conceivable that changes in arterial elastic properties would occur in reaction to hyperemia. However, we have not found literature reporting the arterial biomechanical properties quantitatively with hyperemia.

The hypothesis of the present study is therefore that arterial elasticity would change following reactive hyperemia. This paper therefore describes the investigation of the impact on possible changes in arterial elasticity with hyperemia. We have recently developed a smartphone-based compact and easy-to-operate device capable of noninvasive measurement of elastic properties (volume elastic modulus (*E*_v_) and volume change ratio (*η*_v_)) in finger and radial arteries (see further explanations and overview of the system shown in Figure A2 in the Appendix (a-1)). These measures are expressed as an exponential function of mean blood pressure (*MBP*) or transmural pressure (*P*_tr_ = *MBP* − (externally applied pressure or cuff pressure, *P*_c_))^17^, based on the combination of transmittance- or semi-transmittance-type infrared photo-plethysmography and volume-oscillometric sphygmomanometry (VOS)^17–21^. Therefore, following on from the above background, this paper describes a first approach for studying the impact of changes in elastic properties of these peripheral arteries with reactive hyperemia. This has implications for the possibility of creating a convenient assessment of endothelial activity as a substitute for the FMD test.

## Results

**Figure 1:**
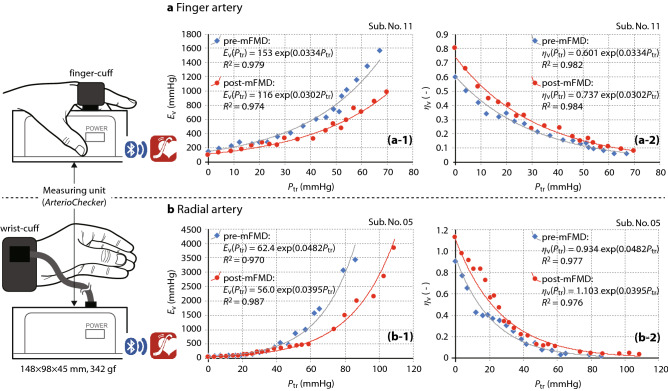
Examples of *E*_v_(*P*_tr_)− *P*_tr_ and *η*_v_(*P*_tr_) − *P*_tr_ curves before (pre-mFMD) and after cuff occlusion (post-mFMD) on the finger and the radial arteries. Two examples of *E*_v_(*P*_tr_) − *P*_tr_ curves (upper left panel; (**a-1**) and lower left; (**b-1**)) and *η*_v_(*P*_tr_) − *P*_tr_ curves (upper right panel; (**a-2**) and lower right; (**b-2**)) before (data points of solid circles; pre-mFMD) and after cuff occlusion (data points of solid squares; post-mFMD) obtained in the finger (**a**, upper part: Sub. No. 11) and the radial artery (**b**, lower part: Sub. No. 05). Schematic illustrations of the measurement scene in the finger (left upper side) and in the wrist (left lower) are included in this Figure. Solid and grey curves indicate approximately exponential curves for *E*_v_(*P*_tr_) and *η*_v_(*P*_tr_) with coefficients of determination *R*^2^ before and after cuff occlusion, respectively. Each exponential equation is also inserted in this graph. *E*_v_ indicates volume elastic modulus; *P*_tr_, transmural pressure; *η*_v_, volume change ratio; mFMD, modified flow-mediated vasodilatation.

Examples of collected data are shown in Fig. 1. This shows: *E*_v_(*P*_tr_) vs *P*_tr_ (upper left panel; (a-1) and lower left; (b-1)) and *η*_v_(*P*_tr_) vs *P*_tr_ relationships (upper right panel; (a-2) and lower right; (b-2)) before (data points of solid circle; pre-mFMD) and after cuff occlusion (data points of solid diamond; post-mFMD). These were obtained in the finger (upper part: Sub. No. 11) and the radial artery (lower part: Sub. No. 05) of two subjects. Schematic illustrations of the measurement scene in the finger (left upper side) and in the wrist (left lower) are included in this Figure. Solid and grey curves indicate approximately exponential curves for *E*_v_(*P*_tr_) and *η*_v_(*P*_tr_) with coefficients of determination, *R*^2^, before and after cuff occlusion, respectively. Each exponential equation is also inset into each Figure. In the examples, five parameters (*η*_v0_, *E*_v0_, *α*, *K*_stif_ and *k*_dist_) for pre- and post-mFMD and %_parameters (%_*η*_v0_, %_*E*_v0_, %_*α*, %_*K*_stif_ and %_*k*_dist_) were as follows (see also Sub. No. 05 and 11 of Table 1):

- Finger_pre-mFMD; (0.601, 153, 0.0334, 5.11 and 1.24)
- Finger_post-mFMD; (0.737, 116, 0.0302, 3.51 and 1.40)
- Finger_%_changes; (22.6, − 24.2, − 9.58, − 31.2 and 12.4)
- Radial_pre-mFMD; (0.934, 62.4, 0.0482, 3.01 and 1.50)
- Radial_post-mFMD; (1.103, 56.0, 0.0395, 2.21 and 1.83)
- Radial_%_changes; (18.8, − 10.2, − 18.0, − 26.4 and 21.8)

**Table 1:** Summary of the values of five parameters together with coefficients of determination (*R*^2^) for pre- and post-mFMD and those for all of the %_parameters calculated from each parameter value acquired throughout the finger and the radial artery elasticity measurements in each subject.

Sub. No	Gender	Age (years)	BMI (kg/m^2^)	Measuring site	*SBP/MBP/DBP* (mmHg)	*PR* (1/min)	*R*^2^_*η*_V0_ (*P*_tr_)	*R*^2^_*E*_V0_ (*P*_tr_)	*η*_V0_ (−)			
							pre/post	pre/post	*η*_V0_pre_	*η*_V0_post_		
01	Male	22	22.3	Finger	147/100/76	91	0.976/0.949	0.993/0.993	0.411	0.662		
				Radial	136/105/91	92	0.948/0.992	0.996/0.965	0.689	0.992		
02	Male	40	24.3	Finger	125/82/61	71	0.958/0.987	0.982/0.979	0.698	0.812		
				Radial	–	–	–	–	–	–		
03	Male	42	24.9	Finger	130/83/69	59	0.962/0.986	0.933/0.994	0.735	0.963		
				Radial	146/97/72	64	0.987/0.987	0.928/0.987	0.808	0.820		
04	Male	44	22.0	Finger	111/83/69	87	0.978/0.973	0.938/0.963	0.678	0.777		
				Radial	–	–	–	–	–	–		
05	Male	44	23.1	Finger	–	–	–	–	–	–		
				Radial	165/113/87	62	0.935/0.976	0.970/0.987	0.935	1.110		
06	Male	47	24.5	Finger	137/94/72	63	0.963/0.959	0.963/0.966	0.472	0.765		
				Radial	143/103/83	63	0.929/0.919	0.964/0.933	0.531	0.773		
07	Male	69	25.0	Finger	127/82/60	70	0.950/0.969	0.955/0.983	0.468	0.667		
				Radial	130/91/71	59	0.941/0.974	0.987/0.996	1.383	1.429		
08	Male	74	24.9	Finger	135/83/57	59	0.990/0.988	0.994/0.958	0.753	0.947		
				Radial	139/88/63	64	0.987/0.940	0.920/0.993	1.396	1.499		
09	Female	20	22.6	Finger	109/63/40	68	0.963/0.959	0.934/0.974	0.473	0.765		
				Radial	99/59/42	71	0.948/0.980	0.948/0.961	0.699	0.701		
10	Female	24	17.5	Finger	101/60/40	68	0.932/0.976	0.968/0.982	0.390	0.689		
				Radial	92/61/45	69	0.992/0.995	0.979/0.988	1.271	1.859		
11	Female	31	22.6	Finger	134/81/54	75	0.982/0.984	0.979/0.974	0.601	0.737		
				Radial	–	–	–	–	–	–		
12	Female	42	19.5	Finger	95/67/52	71	0.984/0.985	0.941/0.998	0.932	0.953		
				Radial	104/78/65	66	0.985/0.993	0.966/0.966	0.948	1.388		
13	Female	47	20.9	Finger	96/65/49	68	0.956/0.990	0.969/0.977	0.689	0.700		
				Radial	112/84/70	62	0.945/0.966	0.917/0.989	0.800	1.108		
14	Female	55	23.1	Finger	146/96/71	74	0.951/0.970	0.976/0.991	0.468	0.667		
				Radial	124/83/63	68	0.980/0.938	0.992/0.995	1.067	1.410		
15	Female	73	19.7	Finger	124/81/60	72	0.984/0.988	0.938/0.967	0.663	0.817		
				Radial	127/91/73	70	0.932/0.945	0.966/0.972	1.150	1.252		
							Finger: Mean		0.602	0.780		
							SD		0.158	0.107		
							SEM		0.042	0.029		
							Radial: Mean		0.973	1.210		
							SD		0.284	0.353		
							SEM		0.082	0.107		

*E*_V0_ (mmHg)		*α* (mmHg^−1^)		*K*_stif_ (−)		*k*_dist_ (−)		%_*η*_V0_	%_*E*_V0_	%_*α*	%_*K*_*stif*_	%_*k*_*dist*_
*E*_V0_pre_	*E*_V0=post_	*α*_pre_	*α*_post_	*K*_stif_pre_	*K*_stif_post_	*k*_dist_pre_	*k*_dist_post_					
151	112	0.0282	0.0272	4,25	3.05	1.31	1.49	61.1	− 25.5	− 3.55	− 28.1	13.7
67.5	63.2	0.0364	0.0357	2.46	2.26	1.69	1.80	44.0	− 6.37	− 1.92	− 8.18	6.51
97.4	88.0	0.0504	0.0396	4.91	3.51	1.26	1.40	16.3	− 9.65	− 21.4	− 28.5	11.4
–	–	–	–	–	–	–	–	–	–	–	–	–
75.5	48.2	0.0453	0.0391	3.42	1.88	1.41	2.14	31.0	− 36.1	− 13.7	− 45.0	51.1
98.0	97.2	0.0403	0.0246	3.95	2.39	1.34	1.72	1.49	− 0.82	− 38.9	− 39.4	28.4
57.5	50.1	0.0519	0.0449	2.98	2.25	1.50	1.80	14.6	− 12.9	− 13.5	− 24.6	19.7
–	–	–	–	–	–	–	–	–	–	–	–	–
–	–	–	–	–	–	–	–	–	–	–	–	–
62.4	56.0	0.0482	0.0395	3.01	2.21	1.50	1.83	18.8	− 10.2	− 18.0	− 26.4	21.8
137	82.2	0.0315	0.0312	4.33	2.57	1.30	1.64	62.1	− 40.0	− 0.95	− 40.8	26.0
89.1	65.1	0.0414	0.0374	3.69	2.43	1.37	1.70	45.6	− 26.9	− 9.66	− 34.0	23.7
145	88.9	0.0325	0.0323	4.70	2.87	1.27	1.53	42.5	− 38.7	− 0.62	− 39.0	20.8
49.2	37.4	0.0519	0.0515	2.55	1.93	1.64	2.08	3.33	− 24.0	− 0.77	− 24.5	26.5
118	96.6	0.0408	0.0331	4.62	3.18	1.29	3.18	25.8	− 18.1	− 18.8	− 31.1	15.4
48.7	43.2	0.0421	0.0385	2.05	1.66	1.95	2.52	7.38	− 11.3	− 8.55	− 19.0	28.8
179	99.7	0.0454	0.0400	8.12	3.99	1.14	1.33	61.7	− 44.3	− 11.9	− 50.8	17.0
80.1	67.0	0.0409	0.0354	3.28	2.37	1.44	2.37	0.29	− 16.3	− 13.4	− 27.6	20.1
141	100	0.0381	0.0376	5.37	3.76	1.23	1.36	76.5	− 28.9	− 1.31	− 29.9	10.9
36.1	26.9	0.0654	0.0638	2.36	1.72	1.73	2.40	46.3	− 25.5	− 2.45	− 27.3	38.1
153	116	0.0334	0.0302	5.11	3.52	1.24	1.40	22.6	− 24.2	− 9.58	− 31.2	12.4
–	–	–	–	–	–	–	–	–	–	–	–	–
44.1	30.6	0.0686	0.0686	3.03	1.84	1.49	2.19	2.25	− 30.6	− 12.1	− 39.2	46.6
44.2	26.7	0.0667	0.0637	2.95	1.70	1.51	2.43	46.4	− 39.6	− 4.50	− 42.3	60.4
63.8	62.0	0.0481	0.0460	3.07	2.85	1.48	1.54	1.60	− 2.82	− 4.37	− 7.14	5.21
70.3	43.4	0.0479	0.0430	3.37	1.89	1.42	2.12	38.5	− 38.3	− 10.2	− 43.8	49.2
128	112	0.0347	0.0327	4.45	3.66	1.29	1.38	42.5	− 12.7	− 4.66	− 17.8	6.69
52.1	36.1	0.0436	0.0524	2.27	1.53	1.79	2.89	32.1	− 30.7	− 2.75	− 32.6	61.6
111	89.2	0.0410	0.0393	4.40	3.33	1.31	1.47	23.2	− 19.6	− 4.19	− 24.4	12.9
40.2	30.0	0.0597	0.0555	2.40	1.66	1.71	2.52	8.87	− 25.4	− 7.03	− 30.8	46.7
114	84.0	0.0421	0.0381	4.48	3.02	1.32	1.58	34.5	− 24.6	− 8.62	− 31.3	19.3
41.0	26.5	0.0107	0.0084	1.31	0.68	0.11	0.28	23.7	12.6	6.77	11.3	13.7
11.0	7.10	0.0029	0.0023	0.35	0.18	0.03	0.07	6.34	3.36	1.81	3.02	3.66
61.5	49.4	0.0487	0.0443	2.86	1.98	1.59	2.14	24.4	− 21.3	− 9.86	− 29.7	34.3
19.9	21.0	0.0102	0.0120	0.61	0.33	0.19	0.40	19.4	12.3	10.5	10.0	17.0
5.74	6.07	0.0029	0.0035	0.18	0.10	0.05	0.12	5.62	3.56	3.03	2.90	4.91

Gender, age, body mass index (BMI), *SBP*/*MBP*/*DBP* and *PR* obtained by the *ArterioChecker* are also presented. See Abbreviations for explanation of symbols.

Table 1 summarizes the values of five parameters (*η*_v0_, *E*_v0_, *α*, *K*_stif_ and *k*_dist_) together with coefficients of determination (*R*^2^) for pre- and post-mFMD, the mean, standard deviation (SD) and standard error of the mean (SEM) values for all %_parameters calculated from the data acquired throughout the finger and the radial elasticity measurements in each subject. Each participant’s gender, age and body mass index (BMI), together with *SBP*/*MBP*/*DBP* and *PR* obtained by the *ArterioChecker*, are also presented. The elasticity measurements of the finger artery of Sub. No. 05 and of the radial artery of Sub. No. 02, No. 04 and No. 11 were not performed due to the subjects’ wishes and no data were listed for them in Table 1.

**Figure 2:**
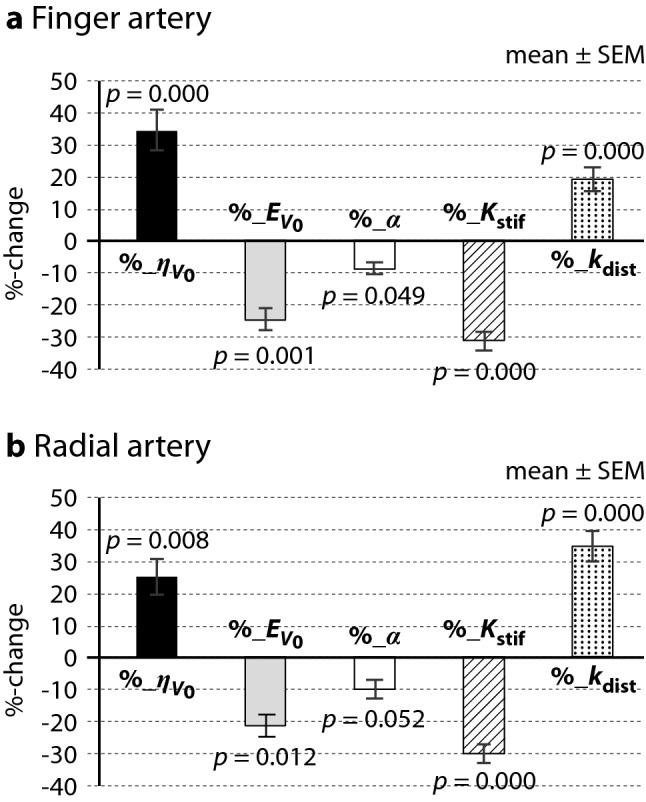
Summarized bar graphs of mean % changes in five parameters in both finger and radial arteries. Bar graphs indicating the mean % changes in five parameters of *η*_v0_, *E*_v0_, *α*, *K*_stif_ and *k*_dist_ (%_*η*_v0_, %_*E*_v0_, %_*α*, %_*K*_stif_ and %_*k*_dist_) obtained in the finger (**a**) and the radial artery (**b**) of all volunteer subjects. Vertical lines in each parameter indicate ± SEM, and the *p*-values of their changes from the baseline are also shown. *η*_v0_ indicates the volume change ratio at *P*_tr_ (transmural pressure) = 0; *E*_v0_, volume elastic modulus at *P*_tr_ = 0; *α*, exponential coefficient related to a stiffness index; *K*_stif_, effective stiffness index of arterial wall; *k*_dist_, distensibility (or vasodilatation) index of arterial wall.

Figure 2 shows bar graphs indicating the mean percentage changes in the five parameters of *η*_v0_, *E*_v0_, *α*, *K*_stif_ and *k*_dist_ (%_*η*_v0_, %_*E*_v0_, %_*α*, %_*K*_stif_ and %_*k*_dist_) obtained in the finger (a) and the radial artery (b) of all volunteer subjects. Vertical lines in each parameter indicate ± SEM, and the *p*-values of their changes from the baseline are also shown. Based on the statistical analysis, *U*-values and 1% or 5% significance levels were obtained respectively to be *U* = 34.0 and *p* < 0.01 (*p* = 0.000) for %_*η*_v0_, *U* = 52.0 and *p* < 0.01 (*p* = 0.001) for %_*E*_v0_, *U* = 70.0 and *p* < 0.05 (*p* = 0.049) for %_*α*, *U* = 28.0, *p* < 0.01 (*p* = 0.000) for %_*K*_stif_, and *U* = 26.0 and *p* < 0.01 (*p* = 0.000) for %_*k*_dist_ in the finger artery, and to be *U* = 42.0 and *p* < 0.01 (*p* = 0.008) for %_*η*_v0_, *U* = 43.5 and *p* < 0.05 (*p* = 0.012) for %_*E*_v0_, *U* = 50.0 and *NS* (*p* = 0.052) for %_*α*, *U* = 12.0 and *p* < 0.01 (*p* = 0.000) for %_*K*_stif_, and *U* = 12.0 and *p* < 0.01 (*p* = 0.000) for %_*k*_dist_ in the radial artery.

## Discussion

The aim of the present study was, first of all, to ask whether expected changes of arterial elastic properties in terms of stiffness and distensibility (or vasodilatation indices) of arterial wall in reaction to hyperemia can be obtained to be consistent with the clinically stated reaction in an FMD test, and, secondly, whether the present method is feasible to be used at home as a convenient assessment method of endothelial activity thereby substituting for such an FMD test.

With respect to the changes of elastic properties both in the finger and in the radial artery measured with the *ArterioChecker*, as shown in an example of Fig. 1, the *E*_v_(*P*_tr_) curves determined approximately as an exponential function of *P*_tr_ in the pre-mFMD test shifted *downwards* in the post-mFMD, indicating the *decrease* of vascular stiffness in reaction to hyperemia over the range of *P*_tr_ regions. On the other hand, the *η*_v_(*P*_tr_) curves in the pre-mFMD shifted *upwards* in the post-mFMD, indicating the *increase* of vascular distensibility (*i.e.*, vasodilatation responses at any *P*_tr_ levels) in reaction to hyperemia. In detail, it was clearly demonstrated, as shown in Fig. 2, that the mean percentage changes of the parameters *η*_v0_ (%_*η*_v0_), *E*_v_ (%_*E*_v0_), *α* (%_*α*), *K*_stif_ (%_*K*_stif_) and *k*_dist_ (%_*k*_dist_) in both finger and radial arteries showed, respectively, a statistically significant *increase*, a statistically *decrease*, a statistically *decrease*, a statistically significant *decrease* and a statistically significant *increase*. As a whole, it should be noted that the *η*_v0_ value, the vasodilatation index in an arterial unloaded state that would be less reflected by smooth muscle^22–24^, showed a significant *increase* (*p* < 0.01), and that the *K*_stif_ value, the stiffness index of the arterial wall, showed a significant *decrease* (*p* < 0.01), while the *k*_dist_ value, the vasodilatation index, showed a significant *increase* (*p* < 0.01) as compared to the baseline in reaction to hyperemia in both finger and radial arteries: In more detail, however, although the parameter values as well as the magnitudes of these percentage changes in increase or decrease varied considerably from person to person, the direction of the increase or decrease of the changes were almost the same, as shown in Table 1 and Fig. 2. These findings suggest that either the finger- or radial-measurement could be adopted to evaluate the vascular endothelial function. It is also noted, however, that the operation of the method by use of the finger measurement is practically easier than that by use of the radial measurement. The reasons for this is that the attachment of the photo-plethysmographic sensor inside the wrist cuff precisely positioned over a radial artery is practically not so easy and required a little getting used to.

Taking these results into consideration, it is clear that peripheral vascular elastic properties in terms of stiffness (or rigidity) and distensibility (or vasodilatation degree) of the vascular wall, composed of elastic and collagen fibres and smooth muscle^22–24^, behave appropriately in reaction to hyperemia. These expected changes are consistent with clinical findings in an FMD test obtained in normal people^3,10^. It should be further noted with this method that the magnitudes of %_*η*_v0_ or %_*E*_v0_ and of %_*α* could be reflected dominantly by the behaviour of elastic fibres and by that of collagen fibres and with smooth muscle, respectively.

It has been recommended that in an FMD test, in order to acquire a reliable assessment of vascular function, it is preferable to make continuous measurements of the brachial arterial diameter, using ultrasonography, over about a 2-min period, or more, until the peak vasodilatation is reached, although the reaction time following the cuff release may differ considerably among individuals^3,10,25^. The effect of this is that the %_FMD varies considerably from person to person, as is similar to the %_parameters determined by the new method. Furthermore, since the elasticity measurement time is approximately 30–40 s, the measurement actually began 1 min after the cuff release. It should therefore be noted that the results obtained by the *ArterioChecker* might include some transient factors in reaction to hyperemia, which might also be one of the causative factors in data variability. Although this drawback due to the measurement technique is unavoidable, it is highly beneficial to be able to assess more reliably the vascular function through the use of various parameters as chosen and reported here.

In this study, the elasticity measurement was carried out in the finger and the radial artery as measuring sites distal from the upper-arm cuff occlusion used to arrest the circulation for the assessment of reactive hyperemia. In contrast, a brachial diameter measurement is performed at a measuring site proximal from the forearm cuff occlusion in a conventional FMD test. This difference in cuff occlusion site may not be a very important issue in assessing the response of reactive hyperemia. This is because, in fact, a peripheral vascular endothelial function test, so-called “reactive hyperemia peripheral arterial tonometry (RH-PAT)”, using a fingertip with photo-plethysmography has also been commonly utilized under the clinical validation through a comparison study with a conventional FMD test^26,27^. Thus, in this respect the present assessment method, *i.e.*, a modified FMD (mFMD) test, would be acceptable as one of the peripheral endothelial function tests.

Photo-plethysmography used in the present elasticity measurement is well known as a simple and convenient means to detect relative volume changes in biological tissue, but it cannot quantify the vascular volume (and its change) and of course the vessel diameter (and its change) as well. Therefore, as mentioned in the Appendix (a-2), the arterial volume change ratio (*η*_v_(*P*_tr_) = Δ*V*_a_/$\overline{V}_{{\text{a}}}$(*P*_tr_)) and arterial volume ratio ($\overline{V}_{{\text{a}}}$(*P*_tr_)/*V*_0_) were also used to explain arterial elastic properties. The arterial volume change ratio (*η*_v_(*P*_tr_)) is related to the normalized pulse volume due to pulse pressure and the arterial volume ratio ($\overline{V}_{{\text{a}}}$(*P*_tr_)/*V*_0_) concerns the mean intra-arterial pressure (*P*)–volume (*V*) relationship (or strictly speaking, dynamic *P*–*V* relationship), which reflects essentially the nonlinear visco-elastic behaviour of a blood vessel itself^20,22–24,28–30^.

**Figure 3:**
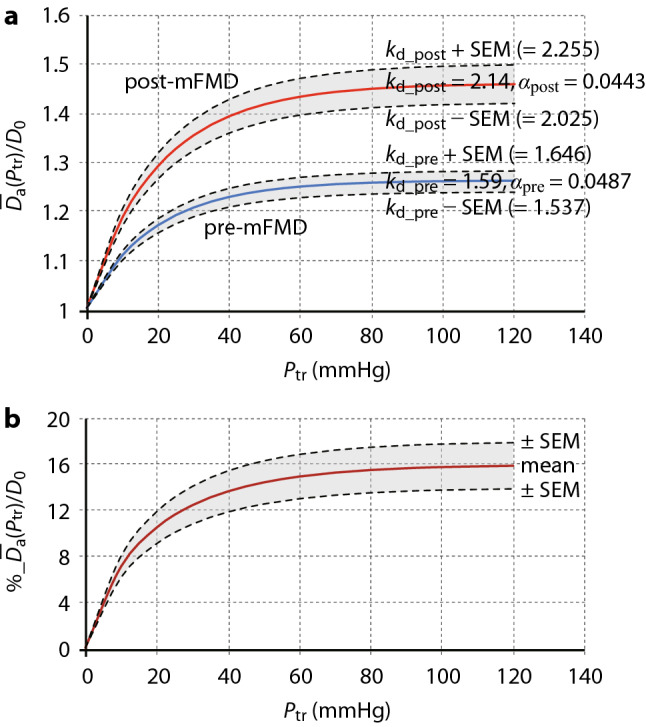
Diameter ratio ($\overline{D}_{{\text{a}}}$(*P*_tr_)/*D*_0_) − *P*_tr_ and %_$\overline{D}_{{\text{a}}}$(*P*_tr_)/*D*_0_ − *P*_tr_ relationships. (**a**) Diameter ratio ($\overline{D}_{{\text{a}}}$(*P*_tr_)/*D*_0_) − *P*_tr_ curves calculated by Eq. (1) using the measured parameters *k*_dist_ and *α* of mean values obtained from all subjects before (pre-mFMD: *k*_d_pre_ = 1.59 (SEM = 0.0546), *α*_pre_ = 0.0487) and after cuff occlusion (post-mFMD: *k*_d_post_ = 2.14 (SEM = 0.115), *α*_post_ = 0.0443). In this graph, the values of *α*_pre_ and *α*_post_ are constant, and the curves with the values of *k*_d_pre_ ± SEM and *k*_d_post_ ± SEM are also shown. (**b**) Mean %_$\overline{D}_{{\text{a}}}$(*P*_tr_)/*D*_0_ ± SEM vs *P*_tr_ relationships calculated from the diameter ratio ($\overline{D}_{{\text{a}}}$(*P*_tr_)/*D*_0_) − *P*_tr_ curves of pre- and post-mFMD. *α*_pre_ *and α*_post_ indicate exponential coefficients before and after occlusion, respectively; *k*_d_pre_ and *k*_d_post_, distensibility indices of arterial wall before and after occlusion, respectively; *P*_tr_, transmural pressure; mFMD, modified flow-mediated vasodilatation; SEM, standard error of the mean.

Taking these points into consideration, we can calculate the pressure (*P*_tr_) − diameter ratio ($\overline{D}_{{\text{a}}}$(*P*_tr_)/*D*_0_) relationship using equation (A-7) (see Appendix (a-2)) when replacing $\overline{V}_{{\text{a}}}$(*P*_tr_)/*V*_0_ (= $\uppi \cdot \overline{D}_{{\text{a}}}^{2}$(*P*_tr_)/4/$\uppi \cdot \overline{D}_{0}^{2}$/4) = $\overline{D}_{{\text{a}}}^{2}$(*P*_tr_)/$\overline{D}_{0}^{2}$) with $\overline{D}_{{\text{a}}}$(*P*_tr_)/*D*_0_ as,

1 $$\overline{D}_{{\text{a}}} (P_{{{\text{tr}}}} )/D_{0} = \surd\,\,(k_{{{\text{dist}}}} {-} \, (k_{{{\text{dist}}}} {-}{ 1})) \cdot {\text{exp}}({-}\alpha \cdot P_{{{\text{tr}}}} )$$

where $\overline{D}_{{\text{a}}}$(*P*_tr_) is arterial diameter at *P*_tr_, and *D*_0_ diameter at *P*_tr_ = 0. Figure 3a shows examples of $\overline{D}_{{\text{a}}}$(*P*_tr_)/*D*_0_ − *P*_tr_ curves calculated by Eq. (1) using the parameters *k*_dist_ and *α* of mean values obtained in the radial artery of all participants *before* (pre-mFMD: the measured mean values were *k*_d_pre_ = 1.59 (SEM = 0.0546), *α*_pre_ = 0.0487) and *after* cuff occlusion (post-mFMD: those were *k*_d_post_ = 2.14 (SEM = 0.115), *α*_post_ = 0.0443). In this graph, the values of *α*_pre_ and *α*_post_ are constant, and the curves with the values of *k*_d_pre_ and *k*_d_post_ as well as those ± SEM are shown. If the *D*_0_ (or *V*_0_) values before and after cuff occlusion are nearly the same, %_$\overline{D}_{{\text{a}}}$(*P*_tr_)/*D*_0_ − *P*_tr_ relationships can also be calculated as shown in Fig. 3b: It is naturally noted that %_$\overline{D}_{{\text{a}}}$(*P*_tr_)/*D*_0_ values are largely dependent upon the distending pressure of the artery, that is, *P*_tr_ or *MBP* when *P*_c_ = 0. The mean %_$\overline{D}_{{\text{a}}}$(*P*_tr_)/*D*_0_ value at higher *P*_tr_ region is about 16% (mean %_$\overline{V}_{{\text{a}}}$(*P*_tr_)/*V*_0_ value is about 25.6%). The value of about 16% increase in the diameter is considered to be comparable to the value of %_FMD (from 6–7 to around 25%) obtained in normal people^3,10^. This agreement seems to support the value of this method for noninvasive assessment of peripheral vascular endothelial function.

The FMD with *Doppler* ultrasound has currently emerged as the most popular clinical method for assessing vascular endothelial function in a noninvasive manner. However, for the accurate and reliable assessment of vascular function it is essential, firstly, to have access to appropriate ultrasonic equipment and this must be in conjunction with the high level of skill required for the precise and continuous measurement of vessel diameter. Further to these technical issues, it is noted that the vessel diameter measured is dependent upon mean blood pressure (*MBP*) at that time: Strictly speaking, it should therefore be considered that the value of %_FMD should be compensated for the same level of *MBP*. Our new method does not need to consider this point and so this is an important feature, because the measures of *E*_v_(*P*_tr_) and *η*_v_(*P*_tr_) can be determined simply as a function of transmural pressure (*P*_tr_ = *MBP* − *P*_c_) and the five parameters of *η*_v0_, *E*_v0_, *α*, *K*_stif_ and *k*_dist_ derived experimentally and analytically are independent of *MBP*.

Compared with a conventional ultrasound assessment of endothelial cell function by an FMD test in the brachial artery, our new method described herein is much simpler and more convenient to use without any advanced skill being required either in the finger or in the radial artery; it is similar to dealing with a home-sphygmomanometer. As mentioned in the Introduction section, if a new technology with similar performance to the FMD test can be used by anyone at home, it could be useful for early detection of vascular dysfunction. Such a technology could also be helpful for a person with early arteriosclerosis to carry out a follow-up examination as part of his/her lifestyle improvement or monitoring their therapeutic drug programme. Further comparison experiments with the FMD test are now needed using a larger number of subjects, including patients with cardiovascular diseases. Nevertheless, the present experimental results obtained at this stage of development show the potential for offering a useful and convenient assessment method for peripheral vascular functional reactivity. This method has a considerable advantage of simultaneous acquisition of both degree of vasodilatation and arterial elasticity as well as of behaviour of elastic and collagen fibres and smooth muscle as components of the vessel wall. It is also important and interesting to investigate the effects of vascular tone due to smooth muscle using a nitroglycerin-mediated dilation with the present method.

## Methods

### Evaluation parameters related to arterial elasticity (see Appendix (a-1)–(a-3))

A brief review of the arterial elastic properties from a biomechanical viewpoint is given in the Appendix (a-1). In this study we adopted the volume elastic modulus (*E*_v_(*P*_tr_)) and the volume change ratio (*η*_v_(*P*_tr_)$$)$$ measured photo-plethysmographically to determine as an exponential function of arterial distending pressure (or transmural pressure (*P*_tr_ = *MBP* − *P*_c_; (*MBP* is calculated by a well-known formula as *DBP* = (3·*MBP* − *SBP*)/2^31^)) as shown in Table 2. The measurement principle based on the photo-plethysmography technique is presented in the Appendix (a-2). Using experimentally and analytically derived measures (see also Appendix (a-2)), the five constant parameters, which were related to elastic property factors and independent of *MBP* (or *P*_tr_), were used as elasticity evaluation indices; these are summarised in Table 2, where physiological interpretations of these parameters are explained in detail (see also Appendix (a-3)).

**Table 2:** Expression of volume change ratio (*η*_v_(*P*_tr_)) and volume elastic modulus (*E*_v_(*P*_tr_)) as an exponential function of transmural pressure (*P*_tr_) derived photo-plethysmographically and used as arterial elastic measures in the present study, with five evaluation parameters related to arterial elasticity and vasodilatation and their physiological interpretations.

Expression of arterial elastic properties	
Volume change ratio	Volume elastic modulus
*η*_v_(*P*_tr_) = *η*_v0_·exp(− *α*·*P*_tr_)	*E*_v_(*P*_tr_) = *E*_v0_·exp(*α*·*P*_tr_)

Evaluation parameters	Physiological interpretations
*η*_v0_ (−)	Volume change ratio at *P*_tr_ = 0: Vasodilatation index in an unloaded state of the artery dominantly reflected by elastic fibres
*E*_v0_ (mmHg)	Volume elastic modulus at *P*_tr_ = 0: Stiffness index in an unloaded state of the artery dominantly characterised by elastic fibres
*α* (mmHg^−1^)	Exponential coefficient: Stiffness index affecting arterial elasticity in higher *P*_tr_ (or *BP*) region dominantly reflected by collagen fibres and smooth muscle
*K*_stif_ (= *E*_v0_·*α*) (−)	Effective stiffness index over the range of *P*_tr_
*k*_dist_ (= *K*_stif_/(*K*_stif_ − 1) (−)	Effective distensibility (or vasodilatation) index over the range of *P*_tr_

### Apparatus

A detailed description of the smartphone-based instrument (named “*ArterioChecker*”) for the finger and the radial artery used in this study has been reported elsewhere^17^. Briefly, for the elasticity measurement in the finger or the radial artery, a finger cuff (width of 30 mm) or a wrist cuff (width of 70 mm) was used, inside of which was embedded a photo-plethysmographic sensor to detect the light intensity (*I*), *i.e.*, photo-plethysmogram (*PPG*): This sensor consisted of a near-infrared light emitting diode (LED: SMC810; peak emission wavelength 810 nm, Epitex Inc., Kyoto, Japan) as a light source and a photo-diode (PD: S6775-01, Hamamatsu Photonics K.K., Hamamatsu, Japan) as a photodetector.

For a finger-measurement, the occlusive cuff was wrapped around the basal phalanx of a right index finger. The LED and the PD were positioned inside the cuff on opposite sides of the finger. Then, for a wrist-measurement, the occlusive cuff was wrapped around the left wrist, inside of which the LED and the PD were fixed 20 mm apart from the center of each element so as for a radial artery to be placed between them.

Bluetooth communication was made between the *ArterioChecker* and the smartphone (iPhone) operated by an experimental app with appropriate software. This was usable by iPhone 7 and later devices with iOS 13.3 or subsequent upgrades (Apple Inc., Cupertino, USA). The program had a number of functions: Firstly, it allowed transmission of all of the analog-to-digital converted signals (cuff pressure *P*_c_ and *PPG* signals) from the *ArterioChecker* to the iPhone. Secondly; it carried out the control of the measurement start/stop process of the iPhone, with real-time display of signal waveforms; Thirdly, it carried out signal processing and calculations of various quantities. These were as follows: *PPG* raw signal, *SBP*/*MBP*/*DBP*, pulse rate (*PR*), *P*_tr_ (*MBP* − *P*_c_), *η*_v_(*P*_tr_) (equation (A-2) in the Appendix (a-2); same as below), *E*_v_(*P*_tr_) (equation (A-3)), $\overline{V}_{{\text{a}}}$(*P*_tr_)/*V*_0_ (equation (A-6)), *K*_stif_ (equation (A-10)) and *k*_dist_ (equation (A-9)). Data were stored in the iPhone and also stored and managed on a cloud server provided for this study. The five parameters *η*_v0_, *E*_v0_, *α*, *K*_stif_ and *k*_dist_ thus obtained were used for the data analysis.

Overview of the elasticity measurement system for the finger and radial artery was given in Figure A2 in the Appendix (a-2).

### Participants and ethics

A total of 15 volunteers (eight males and seven females) participated in this pre-clinical human study. All were Japanese, 20–74 years old, living their normal daily life in Tokyo, Osaka, Kobe and Sapporo cities. Three of the 15 participants (No. 07, No. 08 and No. 15 in the Results section of Table 2) were suffering from grade I hypertension and had been taking antihypertensive agents every day. The other participants from the age of 20 to 55 had no current cardiovascular disease and did not take any prescription medications, although two participants (No. 09 and No. 13), who have had normal daily lives, appeared to have hypotension.

Written informed consent was obtained from the participants after they were provided with a complete description of the study, inclusive of measurement procedures. This study was approved by an institutional review board of the ethics committee of Asahikawa Medical University (reference approval number 19039) and conducted according to the principles expressed in the Declaration of Helsinki. This was not a replicated study.

### Measurement procedures

All experiments were carried out in a room temperature of around 24 to 25 ℃ and humidity of approximately 50%. Throughout the measurements, the participant remained as motionless as possible, sitting for one minute at rest in a chair with his/her left hand placed on a desk at heart level before starting the measurements. A brachial cuff used for both *BP* measurement and arterial occlusion, together with the wrist cuff for the radial elasticity measurement, were attached to the participant’s left upper arm and the wrist, respectively. These, together with the finger cuff for the finger elasticity measurement, were attached to the participant’s right arm and the finger, respectively. Before the experiment in each participant the brachial *BP* was measured by a conventional sphygmomanometer (NISSEI DSK-1051, authenticated by the European Society of Hypertension; Japan Precision Instruments, Inc., Japan), and *SBP*_c_/*DBP*_c_ and pulse rate (*PR*_c_) values were recorded as a reference.

As mentioned in the Introduction section, the usual FMD test starts by measuring the inner diameter of the brachial artery before and after a 5-min supra-systolic cuff occlusion of the forearm. However, since the measuring site was the finger or the radial artery, the brachial cuff occlusion was carried out in this study to allow for the possibility of similar effects of reactive hyperemia in the peripheral vasculature, as would be with peripheral vascular endothelial function testing^26,27^. We will therefore call this a modified FMD (mFMD) test hereafter.

Prior to the elasticity measurement in the finger and the radial artery, three repeated tests were carried out to evaluate the repeatability of the five parameters in each participant. Mean relative errors (percentage changes from the averaged values) of the parameters *η*_v0_, *E*_v0_, *α*, *K*_stif_ and *k*_dist_ were respectively less than 3.0, 5.0, 4.0, 5.0 and 2.0% for the finger and less than 5.0, 5.0, 3.0, 5.0 and 5.0% for the radial artery, indicating acceptable repeatability of each parameter.

The measurement procedures were as follows: After 1 min of rest at the end of *BP* measurement, the radial (or finger) elasticity measurement was made as a control (baseline) condition three times to confirm almost the same *E*_v_(*P*_tr_) and *η*_v_(*P*_tr_) data, obtaining averaged data as a pre-mFMD test. Then, the connection of the brachial cuff was changed from the sphygmomanometer to a handmade cuff-inflation/deflation device and the cuff pressure was increased approximately 50 mmHg higher than individual *SBP*_c_ level during 5 min to completely arrest the circulation in the arm. The rising/falling times of the cuff pressure from 0/200 to 200/0 mmHg were less than 0.5 s. In 1 min after release of the cuff occlusion, the radial (or finger) elasticity measurement was done only once to obtain the data as a post-mFMD test.

After taking a 10 min break at the end of the radial (or finger) elasticity measurement, the finger (or radial) elasticity measurement was carried out with the same procedure as for the radial (or finger) measurement. Note that the order of the finger and the radial measurement was counter-balanced for each individual. If a subject did not wish to proceed with the subsequent supra-systolic cuff occlusion for the elasticity measurement due to discomfort and/or feeling unwell, either the finger or the radial measurement was done accordingly.

### Data analysis

The measured *E*_v_(*P*_tr_) and *η*_v_(*P*_tr_) data that approximated to an exponential function (*i.e.*, *E*_v_(*P*_tr_) = *E*_v0_·exp(*α*·*P*_tr_); *η*_v_(*P*_tr_) = *η*_v0_·exp(− *α*·*P*_tr_)) with greater than 0.9 of the coefficient of determination (*R*^2^), were adopted for analysis in each subject. Actually, it is noted that the *R*^2^ values were over 0.9 in all of the elasticity measurements both in the finger and in the radial artery.

The changes in the five parameters, *η*_v0_, *E*_v0_, *α*, *K*_stif_ and *k*_dist_, as a consequence of the reactive hyperemia, were compared to the baseline parameters and calculated as a percentage of these baseline parameters (%_*η*_v0_ (= ((*η*_v0_post_ − *η*_v0_pre_)/*η*_v0_pre_) × 100), %_*E*_v0_ (= ((*E*_v0_post_ − *E*_v0_pre_)/*E*_v0_pre_) × 100), %_*α* (= ((*α*_post_ − *α*_pre_)/*α*_pre_) × 100), %_*K*_stif_ (= ((*K*_s_post_ − *K*_s_pre_)/*K*_s_pre_) × 100) and %_*k*_dist_ (= ((*k*_d_post_ − *k*_d_pre_)/*k*_d_pre_) × 100)) in each and in all of the subjects. Subscripts “pre” and “post” mean respectively before (baseline) and after cuff occlusion. The statistical significance of the mean percentage changes from the baseline in each parameter obtained in all subjects was also examined by the nonparametric *Mann–Whitney U*-test for the comparison between two conditions, *i.e.*, pre-mFMD and post-mFMD. For data that passed tests, data were expressed as means ± SEM. All *p*-values are two-sided, and *p* < 0.05 and *p* < 0.01 were considered as a statistically significant difference. The analyses were carried out using Microsoft Excel and IBM SPSS Statistics 19.0 (Stats Guild Inc., Chiba, Japan).

### Role of the funding source

The funder of the study had no role in study design, data collection, data analysis, data interpretation, writing of the report, or the decision to submit for publication.

## Supplementary Information

Supplementary Information.

## Data Availability

The datasets used and/or analyzed during the current study are available from the corresponding author on reasonable request (takehiroy@me.com).

## Abbreviations

ABI: Ankle-brachial pressure index
BMI: Body mass index
*D*_a_: Arterial diameter
$\overline{D}_{0}$: Arterial diameter at *P*_tr_ = 0 (diameter in an unloaded state of the artery)
*D**BP*: Diastolic blood pressure
Δ*I*: Pulsatile component of *I*
Δ*P*: Pulse pressure (= *SBP* − *DBP*)
*E*_inc_: Incremental elastic modulus
*E*_p_: Pressure elastic modulus
*E*_v_: Volume elastic modulus
*E*_v0_: Volume elastic modulus at *P*_tr_ = 0 (stiffness index in an unloaded state of the artery)
*E*_v0_post_: *E*_V0_ after cuff occlusion
*E*_v0_pre_: *E*_V0_ before cuff occlusion
FMD: Flow-mediated dilation
*I*: Transmitted or semi-transmitted light intensity
*K*_stif_: Stiffness (or rigidity) index of arterial wall (= *k*_dist_/(*k*_dist_ − 1))
*K*_s_post_: *K*_stif_ after cuff occlusion
*K*_s_pre_: *K*_stif_ before cuff occlusion
*k*_dist_: Distensibility (or vasodilatation) index of arterial wall (= *V*_max_/*V*_0_))
*k*_d_post_: *k*_dist_ after cuff occlusion
*k*_d_pre_: *k*_dist_ before cuff occlusion
LED: Light emitting diode
mFMD: Modified flow-mediated dilation
*M**BP*: Mean blood pressure
*P*_c_: Cuff pressure
PD: Photo-diode
*PPG*: Photo-plethysmogram
*PR*: Pulse rate
*P*_tr_: Transmural pressure of the arterial wall
PWV: Pulse wave velocity
*R*^2^: Coefficient of determination
*S**BP*: Systolic blood pressure
SEM: Standard error of the mean
SD: Standard deviation
*V*_a_: Arterial volume
*V*_max_: Arterial volume when $P_{{{\text{tr}}}} \, \to \,\infty$
VOS: Volume-oscillometric sphygmomanometry
*V*_0_: Arterial volume in an unloaded state
*α*: Exponential coefficient
*α*_post_: *α* after cuff occlusion
*α*_pre_: *α* before cuff occlusion
*η*_v_: Volume change ratio
*η*_v0_: Volume change ratio at *P*_tr_ = 0 (vasodilatation index in an unloaded state of the artery)
*η*_v0_post_: *η*_V0_ after cuff occlusion
*η*_v0_pre_: *η*_V0_ before cuff occlusion
