# Supplementary material for: Peripheral arterial elasticity changes derived by volume-oscillometry in reaction to hyperemia as a possible assessment of flow-mediated vasodilatation
Source: Sci Rep. 2022 Nov 14;12:19479. doi: 10.1038/s41598-022-22050-1 (PMC9663529; doi:10.1038/s41598-022-22050-1)
Supplement: Supplementary file 1 — Supplementary Information. [file 41598_2022_22050_MOESM1_ESM.pdf]

Appendix

(a-1) On the arterial elastic property

Acquisition of arterial elasticity is of great importance for diagnosis and treatment of cardiovascular related diseases. It is well known from a biomechanical viewpoint that there are mainly three representative measures that can reflect the dynamic elastic properties of the arterial wall, characterized by its composition. This includes elastic and collagen fibres and smooth muscle[S1,S2,S3]. If an artery is assumed to be cylindrical, then the three measures of interest are (i) incremental elastic modulus (E_{inc}), defined as $E_{inc} = \Delta P \cdot r/h/\Delta r/r = \Delta P \cdot D^2/2h \cdot \Delta D$, (ii) pressure elastic modulus (E_p), as $E_p = \Delta P/\Delta D/D$ and (iii) bulk or volume elastic modulus (E_v), as $E_v = \Delta P/\Delta V/V$. As can be seen, these moduli have the following mutual relationships as: $E_{inc} = (D/2h) \cdot E_p = (D/h) \cdot E_v$, where ΔP; pulse pressure, r; arterial radius, Δr; radius change due to ΔP, D (= $2r$); arterial diameter, ΔD; diameter change due to ΔP and h; wall thickness. It should be noted that these moduli have a strong dependence on mean arterial distending pressure, *i.e.*, mean blood pressure (*MBP*) or transmural pressure ($P_{tr} = MBP - P_c$), caused by a nonlinearity of the pressure (P; *MBP* or P_{tr}) – volume (V) curve in an artery[S1,S4,S5,S6] (see Figure A1): The moduli at lower and higher P regions mainly reflect the properties of elastic fibres and collagen fibres, respectively[S3] (see also Figure A1).

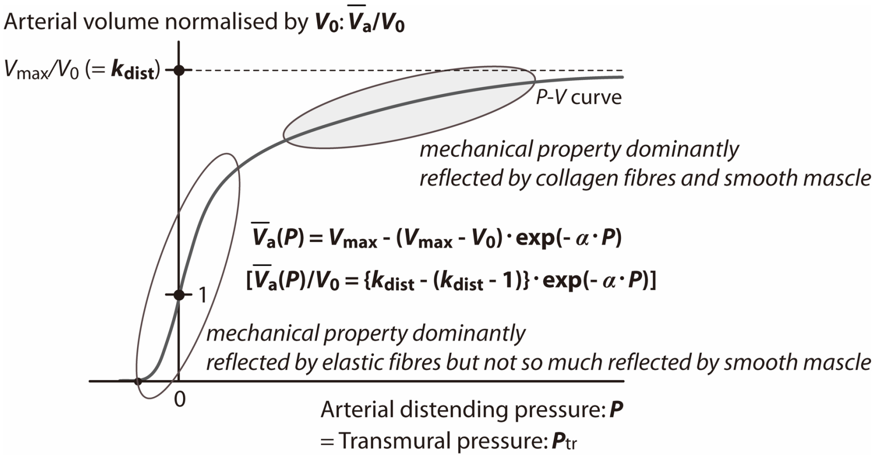

Figure A1. Schematic diagram of inner-arterial pressure (P) – volume (V) relationship (arterial tube law). The P–V curve can be expressed by an exponential function as indicated in the insert. The ordinate denotes arterial volume ($\overline{V}_a$) normalized by an unloaded volume at P = 0 (V_0), the abscissa denotes arterial distending pressure (P; mean blood pressure (*MBP*) or transmural pressure (P_{tr})). α indicates exponential coefficient related to a stiffness index; V_{max}, value of $\overline{V}_a$ when $P_{tr} \rightarrow \infty$; k_{dist} (= V_{max}/V_0, $k_{dist} > 1$), distensibility (or vasodilatation) index of arterial wall.

It is also noted that the arterial dimensions of r, Δr (or D, ΔD) and h are needed to obtain these two moduli except for E_v, which could also be suitable for the evaluation of elasticity in the vascular system. Taking such advantages into consideration, we have previously proposed a noninvasive method for the measurement of E_v in the context of vascular elasticity in the human finger[S7]. Just recently we have devised a convenient smartphone-based instrument capable of real-time E_v measurements at various P_{tr} levels, both in the finger artery and in the superficial radial artery[S8], as shown in Figure A2. This has been found to have, essentially, acceptable accuracy having a high linear correlation as compared with the E_v values determined from a PWV method[S7,S8]. The specially designed instrument (main unit) is named "*ArterioChecker*" in this study.

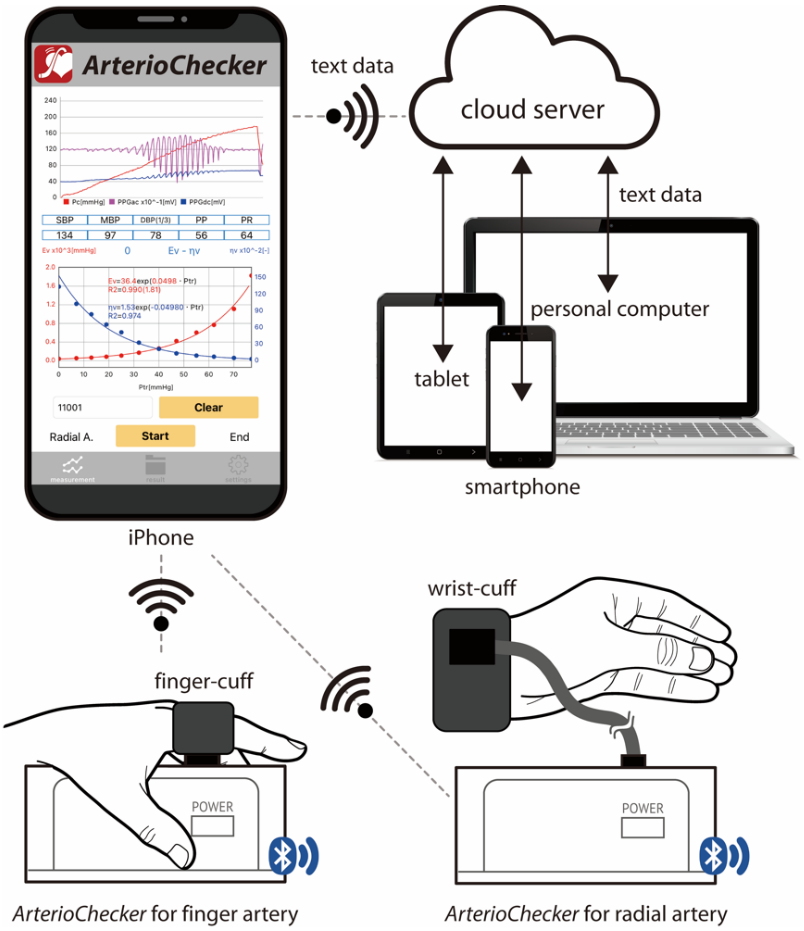

Figure A2. Overview of the smartphone-based peripheral arterial elasticity measurement system. The main unit, named as *ArterioChecker*, is controlled by an iPhone where data display and storage as well as data transmission to the cloud server are also carried out in a fully automatic manner. Data stored on the cloud server can be viewed from a smartphone, tablet and/or personal computer.

(a-2) Brief description of measurement principle

A detailed description of the measurement principle was reported elsewhere[S7,S8]. Briefly, this method is based on the simultaneous measurement of arterial volume change ratio ($\eta_v = \Delta V_a/\bar{V}_a$; where $\bar{V}_a$ is mean arterial volume and ΔV_a is pulse volume superimposed on $\bar{V}_a$) in a chosen biological segment, determined by transmittance- or semi-transmittance-type photo-plethysmography and pulse pressure (ΔP) obtained by volume-oscillometric sphygmomanometry (VOS)[S4,S9,S10]. The E_v can be calculated as $E_v = \Delta P/\eta_v$. In VOS, during the application of cuff pressure (P_c) systolic (SBP) and mean blood pressure (MBP) can be determined and then diastolic BP (DBP) is calculated by a well-known formula as, $DBP = (3 \cdot MBP - SBP)/2$[S11], obtaining ΔP (= $SBP - DBP$). As a whole, η_v and E_v can be acquired as a function of transmural pressure (P_{tr} (= $MBP - P_c$): $\eta_v(P_{tr})$ and $E_v(P_{tr})$).

The $\eta_v(P_{tr})$ can be computed photo-plethysmographically under the assumption that *Lambert-Beer's* law (or modified *Lambert-Beer's* law in consideration of attenuation due to light scattering) holds in the segment (for details see References section (S7, S8)). Theoretically, the $\eta_v(P_{tr})$ is expressed by the following equation:

$$\eta_v(P_{tr}) = \log(1 - \Delta I(P_{tr})/\bar{I}(P_{tr}))/\log(\bar{I}(P_{tr})/I_t) \qquad \text{(A-1)}$$

where $\bar{I}(P_{tr})$ and $\Delta I(P_{tr})$ are the mean and the pulsatile component (in association with pulsatile blood volume change) of the transmitted light intensity ($I(P_{tr}) = \bar{I}(P_{tr}) \pm \Delta I(P_{tr})$), and I_t is the bloodless component of the light intensity, *i.e.*, the light intensity when $P_c \geq SBP$. Following the change (usually 'increase') in P_c, that is, the VOS operation process, MBP and SBP can be obtained and concurrently $\eta_v(P_{tr})$ is determined by equation (A-1) on a beat-by-beat basis, thus calculating the E_v values at various P_{tr} levels "$E_v(P_{tr})$ (= $\Delta P/\eta_v(P_{tr})$)". It has been demonstrated through our experimental results, that $\eta_v(P_{tr})$ and $E_v(P_{tr})$ can be expressed as an exponential function as follows:

$$\eta_v(P_{tr}) = \eta_{v0} \cdot \exp(-\alpha \cdot P_{tr}) \qquad \text{(A-2)}$$

$$E_v(P_{tr}) = E_{v0} \cdot \exp(\alpha \cdot P_{tr}) \qquad \text{(A-3)}$$

where η_{v0}, E_{v0} and α are constants independent of MBP or P_{tr} and are determined as individual arterial elastic properties[S8]. In addition, an arterial volume ratio of $\bar{V}_a(P_{tr})$ and the volume in an unloaded state V_0, which corresponds to the mean volume when $P_{tr} = 0$, this ratio ($\bar{V}_a(P_{tr})/V_0$) can also be calculated as,

$$\bar{V}_a(P_{tr})/V_0 = \log(\bar{I}(P_{tr})/I_t)/\log(\bar{I}(0)/I_t) \qquad \text{(A-4)}$$

We can, thereby, deduce the relationship between $\bar{V}_a(P_{tr})/V_0$ and P_{tr}. This is referred to as the "arterial tube law". Experimentally, as shown in Figure A1, this relationship can also be expressed as,

$$\bar{V}_a(P_{tr}) = V_{max} - (V_{max} - V_0)\cdot\exp(-\alpha\cdot P_{tr}) \qquad \text{(A-5)}$$

Or dividing by V_0, then further rewritten as,

$$\bar{V}_a(P_{tr})/V_0 = V_{max}/V_0 - (V_{max}/V_0 - 1)\cdot\exp(-\alpha\cdot P_{tr})$$

$$= (k_{dist} - (k_{dist} - 1))\cdot\exp(-\alpha\cdot P_{tr}) \qquad \text{(A-6)}$$

where V_{max} and k_{dist} (= V_{max}/V_0; $k_{dist} > 1$) are constant parameters, and V_{max} is the value of $\bar{V}_a$ when $P_{tr} \rightarrow \infty$. In Figure A1, the pressure symbol "P" is used instead of P_{tr}. The derivative of $\bar{V}_a$ with respect to P_{tr} yields $d\bar{V}_a(P_{tr})/dP_{tr} = \alpha\cdot(V_{max} - V_0)\cdot\exp(-\alpha\cdot P_{tr})$, and when we replace "$d$" with "Δ", the following equation can be obtained using equation (A-5) or (A-6) as,

$$E_v(P_{tr}) = \Delta P/(\Delta V_a/\bar{V}_a) = \alpha^{-1}\cdot[(k_{dist}/(k_{dist} - 1))\cdot\exp(\alpha\cdot P_{tr}) - 1] \qquad \text{(A-7)}$$

where ΔP means pulse pressure. Assuming that $(k_{dist}/(k_{dist} - 1))\cdot\exp(\alpha\cdot P_{tr}) \gg 1$, equation (A-7) can be approximated as,

$$E_v(P_{tr}) \fallingdotseq \alpha^{-1}\cdot(k_{dist}/(k_{dist} - 1))\cdot\exp(\alpha\cdot P_{tr})$$

$$= E_{v0}\cdot\exp(\alpha\cdot P_{tr}) \qquad \text{(A-8)}$$

From this equation, the constant k_{dist} can be calculated as,

$$k_{dist} = E_{v0}\cdot\alpha/(E_{v0}\cdot\alpha - 1) \qquad \text{(A-9)}$$

Or rewritten as,

$$E_{v0}\cdot\alpha = K_{stif} = k_{dist}/(k_{dist} - 1) \qquad \text{(A-10)}$$

(a-3) Physiological interpretations of elastic parameters

In the above equations, derived analytically based on experimental findings, the constant parameters, which are related to elasticity factors and independent of *BP*, have the following physiological meanings, taking arterial wall compositions into consideration[S1,S3] (see Figure A1):

η_{v0}; volume change ratio at $P_{tr} = 0$ is given as the maximal pulse volume change ratio in an unloaded state in association with pulse pressure, meaning a distensibility (or vasodilatation) index inclusive of elastic fibres and smooth muscle of arterial wall in

the unloaded state of the artery. In particular, this index would be dominantly reflected by elastic fibres.

E_{v0}; volume elastic modulus at $P_{tr} = 0$ is a stiffness (or rigidity) factor including elastic fibres and smooth muscle in the unloaded state of the artery, which affects arterial elasticity in lower *BP* regions dominantly characterized by elastic fibres.

α; this is an exponential coefficient related to a stiffness (or rigidity) index inclusive of collagen fibres and smooth muscle, which affects arterial elasticity in higher *BP* regions.

K_{stif}; this measure is related to an effective stiffness (or rigidity) index of arterial wall over the range of distending pressure of the artery.

k_{dist}; this measure is related to a distensibility (or vasodilatation) index of the arterial wall over the range of distending pressure of the artery.

References

S1. Gosling, R. G. & Budge, M. M. Terminology for describing the elastic behavior of arteries. *Hypertension* **41**, 1180–1182. https://doi.org/10.1161/01.HYP.0000072271.36866.2A (2003).

S2. Bergel, D. H. The static elastic properties of the arterial wall. *J. Physiol. (Lond.)* **156**, 445–457. https://doi.org/10.1113/jphysiol.1961.sp006686 (1961).

S3. Bergel, D. H. The dynamic elastic properties of the arterial wall. *J. Physiol. (Lond.)* **156**, 458–469. https://doi.org/10.1113/jphysiol.1961.sp006687 (1961).

S4. Yamakoshi, K., Shimazu, H., Shibata, M. & Kamiya, A. New oscillometric method for indirect measurement of systolic and mean arterial pressure in the human finger. Part 1: Model experiment. *Med. Biol. Eng. Comput.* **20**, 307–313. https://doi.org/10.1007/BF02442797 (1982).

S5. Raamat, R., Talts, J., Jagomagi, K. & Lansimies, E. Mathematical modelling of non-invasive oscillometric finger mean blood pressure measurement by maximum oscillation criterion. *Med. Biol. Eng. Comput.* **37**, 784–788. https://doi.org/10.1007/BF02513382 (1999).

S6. Baker, P. D., Westenskow, D. R. & Kuck, K. Theoretical analysis of non-invasive oscillometric maximum amplitude algorithm for estimating mean blood pressure. *Med. Biol. Eng. Comput.* **35**, 271–278. https://doi.org/10.1007/BF02530049 (1997).

S7. Shimazu, H., Yamakoshi, K. & Kamiya, A. Noninvasive measurement of the volume elastic modulus in finger arteries using photoelectric plethysmography. *IEEE Trans. Biomed. Eng.* **33**, 795–798. https://doi.org/10.1109/TBME.1986.325906 (1986).

S8. Yamakoshi, T., Rolfe, P., Kamiya, A. & Yamakoshi, K. Volume elastic modulus with exponential function of transmural pressure as a valid stiffness measure derived by photoplethysmographic volume-oscillometry in human finger and radial arteries: Potential for arteriosclerosis screening. *Med. Biol. Eng. Comput.* **59**, 1585–1596. https://doi.org/10.1007/s11517-021-02391-1 (2021).

S9. Yamakoshi, T. *et al.* Potential for health screening using long-term cardiovascular parameters measured by finger volume-oscillometry: Pilot comparative evaluation in regular and sleep-deprived activities. *IEEE J. Biomed. Health Inform.* **18**, 28–35. https://doi.org/10.1109/JBHI.2013.2274460 (2014).

S10. Yamakoshi, K., Shimazu, H., Shibata, M. & Kamiya, A. New oscillometric method for indirect measurement of systolic and mean arterial pressure in the human finger. Part 2: Correlation study. *Med. Biol. Eng. Comput.* **20**, 314–318. https://doi.org/10.1007/BF02442798 (1982).

S11. Folkow, B. & Neil, E. *Circulation* 1st edn. (Oxford University Press, 1971).
